# Evaluation of Traumatic Brain Injury Severity Using the Abbreviated Injury Scale and the Injury Severity Score: A Retrospective Study in Two Eastern European Centers

**DOI:** 10.3390/jcm14176259

**Published:** 2025-09-04

**Authors:** Iulia-Maria Vadan, Diana Grad, Stefan Strilciuc, Alina Vasilica Blesneag, Marcin Michalak, Vitalie Vacaras, Adina Stan, Dafin F. Muresanu

**Affiliations:** 1 Department of Neurosciences, Iuliu Hatieganu University of Medicine and Pharmacy, 400347 Cluj-Napoca, Romania; alinablesneag@yahoo.com (A.V.B.); vvacaras@umfcluj.ro (V.V.); adinadora@yahoo.com (A.S.); dafinm@ssnn.ro (D.F.M.); 2 RoNeuro Institute for Neurological Research and Diagnostic, 400364 Cluj-Napoca, Romania; diana.grad@brainscience.ro (D.G.); stefan.strilciuc@brainscience.ro (S.S.); 3 Department of Genomics, MEDFUTURE Institute for Biomedical Research, “Iuliu Hațieganu” University of Medicine and Pharmacy, 400012 Cluj-Napoca, Romania; 4 Neurology Clinic, Cluj County Emergency Clinical Hospital, 400347 Cluj-Napoca, Romania; 5 Department of Stroke and Neurology, Saint Vincent’s Hospital, 81-348 Gdynia, Poland; michalak83@gmail.com

**Keywords:** traumatic brain injury, eastern Europe, abbreviated injury scale, AIS, injury severity scale, ISS, trauma severity scoring, retrospective study, neurotrauma epidemiology, modified Marshall score

## Abstract

**Introduction**: Traumatic brain injury (TBI) is a significant global public health issue, with long-term impacts on patients. This study examines the relationship between TBI severity, as measured by the Abbreviated Injury Scale (AIS) and the Injury Severity Score (ISS) at admission, and various sociodemographic, clinical, and injury-related factors. **Methods**: We conducted a retrospective analysis using data from 164 adult TBI patients. All were admitted between March 2020 and June 2023 to two Eastern European tertiary hospitals. Variables included sex, age, education, employment, marital status, injury type and cause, place of injury, and clinical measures such as the Marshall score, AIS, and ISS. Statistical methods included Pearson’s Chi-squared, Fisher’s exact, Spearman correlation, Wilcoxon, and Kruskal–Wallis tests. **Results**: Most patients were male (65.9%), retired (59.8%), and urban residents (73.8%), with a mean age of 64.98 years. The most frequent mechanism of injury was falls (76.2%), typically occurring at home (61%). The predominant injury type was closed head trauma (93.3%). Most patients had mild AIS scores (75%), and the mean ISS was 6.52 (SD: 4.55). Statistically significant group differences were found for AIS among categories of Modified Marshall Score, injury type, and education categories and for ISS among categories of the Modified Marshall Score, injury type, cause and place of injury, employment status, and sex. No significant correlations were found between AIS or ISS and age or hospital length of stay. **Conclusions**: AIS is more anatomically focused. ISS reflects broader systemic injury patterns and is more influenced by contextual factors. These findings are particularly relevant for the Eastern European population and can help develop better healthcare policies for the region.

## 1. Introduction

Traumatic brain injury (TBI) remains a significant healthcare problem worldwide with a high incidence, complex nature, and long-term consequences on the patient’s functionality and quality of life. Approximately half of the world’s population will experience a TBI during their lifetime [1,2]. More than 50 million people currently suffer from TBI [3,4], with 1.5 million admitted annually to the hospitals in the European Union [5]. TBI is also a leading cause of death globally [6].

Many studies have evaluated the most effective scales for assessing TBI severity. Although the Glasgow Coma Scale (GCS) is widely described, other tools must be taken into account [7,8,9,10,11]. The Abbreviated Injury Scale (AIS) is another instrument developed by the Association for the Advancement of Automotive Medicine (AAAM). It represents an anatomical scoring system that ranks each anatomic region on a scale from 1 (minor) to 6 (maximal) using operative findings or neuroradiology [11,12,13,14]. 

The Injury Severity Score (ISS) is a widely used indicator of overall injury severity. It is useful both as an indicator for patient triage and as a clinical measure of injury lethality. ISS is calculated as the sum of the squares of the three highest AIS severity code values [13,15,16]. AIS, compared to the ISS, needs more specifically trained staff due to its more complex nature [13]. 

Researchers continue to seek the best score to predict trauma mortality. ISS is limited because it is restricted to one injury per body part, and can underestimate the severity of the patient’s injuries [17]. 

While AIS is essential for region-specific injury description, ISS may better reflect the overall injury burden and emergency care needs for polytrauma, because it addresses the cumulative effects of injuries that AIS might miss [18].

In this new direction of research, a new and improved ISS score, the NISS (New ISS), has been developed. It is defined as the sum of the squares of the three highest AIS scores overall. Results so far are mixed, and some articles consider it better than the original, while others find no differences between the results [17,19,20,21,22]. More research is needed, as trauma places a substantial burden on global healthcare systems.

Research focusing on Eastern European populations is limited [23,24]. Demographic patterns, healthcare systems, and injury mechanisms may differ from those in Western countries. Studies show that the highest incidence of moderate and severe TBIs was found in Eastern and Central Europe [25]. There is also significant variation in injury-related deaths across Europe. Injury mortality rates are three times higher in Eastern Europe (80/100,000) compared to Western Europe (27/100,000) and twice as high compared to Central Europe (80/100,000) [26]. 

This study examined the correlations and between-group differences in ISS and AIS scores at admission. It focused on various sociodemographic, clinical, injury-related, and hospital-related variables in patients with TBI from two hospitals in Eastern Europe. 

## 2. Methods

We conducted a retrospective observational study using data from 164 TBI patients, aged over 18 years. Data was collected from two hospitals: Cluj County Emergency Hospital in Romania and Saint Vincent Hospital in Gdynia, Poland. The collection period was from March 2020 to June 2023. 

We only analyzed anonymized data of TBI patients without missing data for all the variables of interest (total patients = 180; excluded patients = 16). 

We analyzed sociodemographic variables, injury-related variables, and clinical variables. Sociodemographic variables included age (continuous), sex (male and female), and education (primary school (1–4 years), secondary school (5–8 years), high school (9–12 years), university (13+ years), no formal education, and unknown). Employment type (employed, unemployed, retired, other, unknown), marital status (single, married, living together, separated, widowed, unknown), and residence (rural and urban) were also assessed. Injury-related variables included place of injury (home, other, sports facility, street, and work), cause of injury (aggression, fall, road traffic accident, and other), and type of injury (closed, crushed, and penetrating). Clinical variables were Modified Marshall Score (I–V), comorbidities count (zero, one, two, three, more than three, unknown), Abbreviated Injury Scale (AIS: Mild 2, Moderate 3, Severe 4–5), and Injury Severity Score (ISS). We also analyzed the length of hospital stay (continuous variable). The variables of interest were AIS and ISS. They were determined at admission.

We report descriptive statistics: counts and percentages are given for categorical variables (sociodemographic, clinical and injury-related variables) for the entire sample and stratified by AIS. Mean and standard deviation are provided for continuous variables (age, hospital length of stay, ISS). Mean ISS values with corresponding standard errors are graphed by categorical variables.

To compare the distribution of AIS categories among categorical variables, we used Fisher’s exact test (for education, employment type, marital status, residence, place of injury, cause of injury, type of injury, and Modified Marshall Score) and Pearson’s Chi-squared test (sex). To compare the distribution for ISS among categorical variables, we employed the Wilcoxon’s test (for two-group comparisons) and the Kruskal–Wallis test (for multiple-group comparisons). Post hoc pairwise comparisons were performed using Bonferroni correction to account for multiple comparisons.

Spearman’s correlation was employed to assess correlations between continuous variables (age and hospital length of stay) and AIS categories and ISS scores. The alpha level was set at *p* < 0.05. The descriptive statistics and statistical tests were performed using R v4.3.3. We used the ggplot2 R package (https://cran.r-project.org/web/packages/ggplot2/index.html accessed on 26 June 2025) to create figures.

## 3. Results

### 3.1. Descriptive Statistics of TBI Patients

Of the total of 164 TBI patients, most were male (65.9%), had university as their highest education (42.1%), were retired (59.8%), married (44.5%) and lived in urban areas (73.8%). Most injuries resulted from falls (76.2%), occurring at home (61.0%). Closed injuries were common (93.3%). Most patients had a mild AIS—2 (75%), a Modified Marshall Score II (81.1%), and one comorbidity (27.4%). Additional descriptive statistics for the other variable categories are given in Table 1.

The average hospital length of stay was 8.88 days (SD: 5.56), the average age was 64.98 years (SD: 19.66), and the ISS score averaged 6.52 (SD: 4.66). When stratified by AIS category, the highest mean length of stay (12.9) and age (69) was found among patients with severe AIS 4–5. Age and length of stay for mild and moderate AIS are listed in Table 2.

**Figure 1:**
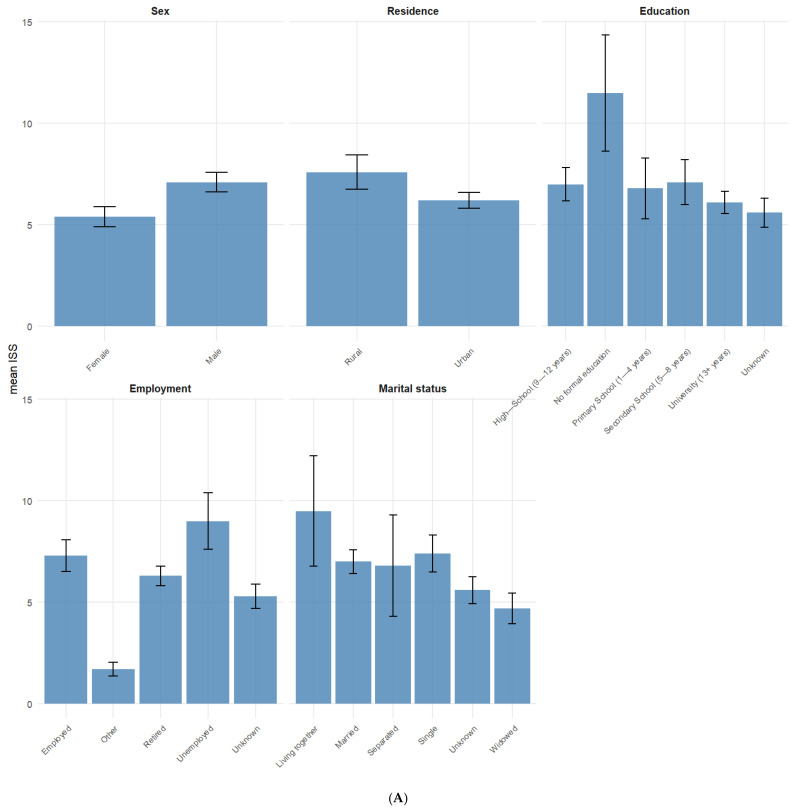

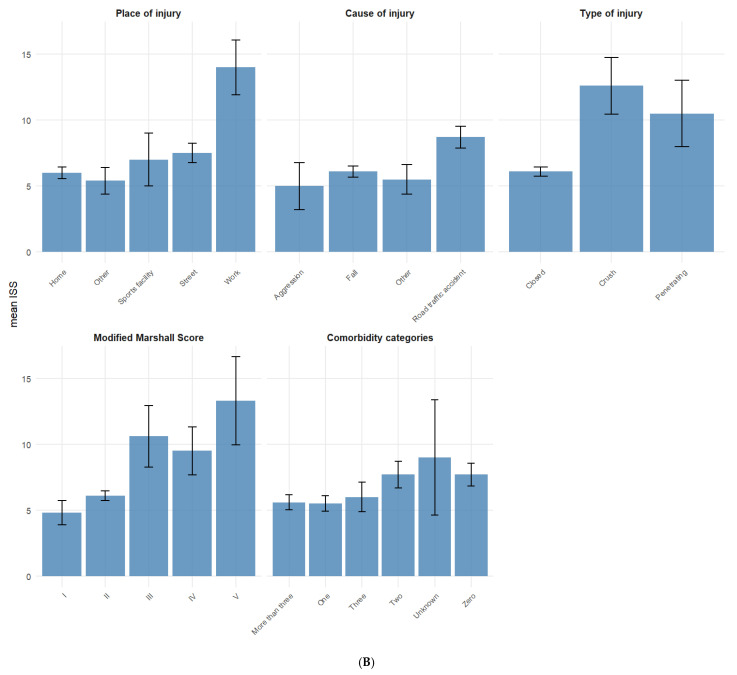
(**A**,**B**) Mean ISS for categorical variables. Error bars represent standard error of the mean ISS.

The mean ISS stratified across different categorical variables (Figure 1) was highest for those who sustained the TBI at work (14) and those with a Modified Marshall Score V (13.3) while the lowest mean ISS was found among TBI patients that listed “other” as their place of employment (1.7) and those who were widowed (4.7).

**Figure 2:**
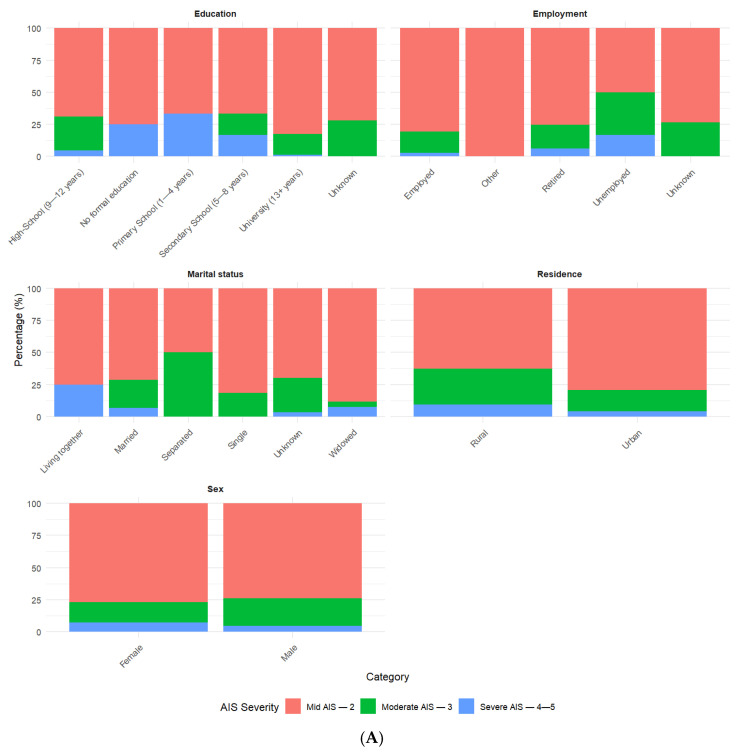

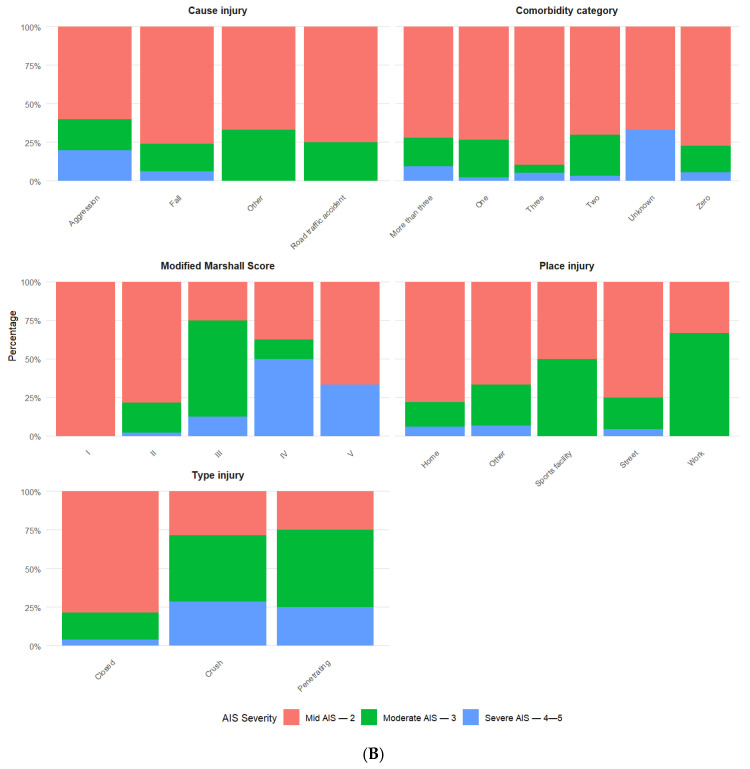
(**A**,**B**) Percentages for categorical variables by AIS categories.

Mild AIS—2 was the predominant category for most socio-demographic and injury-related variables (Figure 2). There were a few exceptions, such as Modified Marshall Score III, type of injury (crush and penetrating), and place of injury (work), with Moderate AIS—3 being lesspredominant. For Modified Marshall Score IV, Severe AIS—4–5 was more predominant.

### 3.2. AIS Results

Based on Fisher’s exact test, we found statistically significant differences for AIS and the Modified Marshall Score (*p*-value < 0.001), type of injury (*p*-value = 0.001), and education (*p*-value = 0.020). Pairwise group comparison showed statistically significant differences only between those with a Modified Marshall Score of I and IV (adjusted *p*-value = 0.036), of II and III (adjusted *p*-value = 0.022), and of II and IV (adjusted *p*-value = 0.002). For type of injury, differences were only found among those with crush and closed injury (adjusted *p*-value = 0.015). No statistically significant pairwise comparisons were found for education.

For other analyzed variables, there were no statistically significant differences for marital status (*p*-value = 0.135), residence (*p*-value = 0.071), cause of injury (*p*-value = 0.369), place of injury (*p*-value = 0.384), employment status (*p*-value = 0.455), and comorbidity categories (*p*-value = 0.404) Post hoc pairwise comparisons showed no significant results as well. For sex, there were no statistically significant differences for AIS (*p*-value = 0.613) based on Pearson’s Chi-squared test.

Kruskal–Wallis test results showed no statistically significant differences in age (χ^2^(2) = 0.45, *p*-value = 0.797) and length of stay (χ^2^(2) = 4.92, *p*-value = 0.085) among the AIS categories.

### 3.3. ISS Results

Spearman correlations showed a weak negative, not statistically significant correlation between ISS and age (rho = −0.142, *p*-value = 0.068) and a weak positive, not statistically significant correlation (rho = 0.101, *p*-value= 0.195) for ISS and the length of stay. Wilcoxon test results showed statistically significant group differences only for sex (W = 2433, *p*-value 0.039), but not for residence (W = 2886, *p*-value = 0.285). 

The Kruskal–Wallis test showed statistically significant ISS group differences for the Modified Marshall Score (χ^2^(2) = 13.97, *p*-value = 0.007), type of injury (χ^2^(2) = 12.06, *p*-value = 0.002), employment status (χ^2^(2) = 11.43, *p*-value = 0.022), cause of injury (χ^2^(2) = 10.97, *p*-value = 0.012), and place of injury (χ^2^(2) = 10.71, *p*-value = 0.030). 

Post hoc pairwise comparison showed statistically significant differences for the following: (a) injury type, between those with crush and closed injuries (*p* = 0.004); (b) injury cause, between road traffic and falls (*p* = 0.003); (c) place of injury, between home and at work (*p* = 0.041); and (d) employment, between those employed and other (adjusted *p* = 0.047), and between those unemployed and other (*p* = 0.010). No statistically significant pairwise differences were found between any of the Modified Marshall Score categories (*p* = 0.003). 

The differences in ISS among categories for marital status (χ^2^(2) = 8.53, *p*-value = 0.129) education (χ^2^(2) = 5.06, *p*-value = 0.409), and comorbidity category (χ^2^(2) = 5.00, *p*-value = 0.415) were not statistically significant. No statistically significant pairwise comparisons were found for marital status and education either.

## 4. Discussions

The current research was a retrospective study. It explored the relationship between injury severity, measured by the Abbreviated Injury Scale (AIS) and Injury Severity Score (ISS), and a variety of socio-demographic, injury-related, and clinical factors in a cohort of 164 TBI patients from two tertiary care units from Eastern Europe, one in Romania and one in Poland.

Similarly to other literature [23,27,28,29], most patients in our sample were male (65.9%) and older, with a mean age of 65 years. The most frequent causes of TBI were falls (76.2%), followed by road traffic accidents (17.1%). These results are consistent with other research [28,30,31,32,33], where falls were the primary cause of injury, especially in geriatric patients. Closed injuries and low-to-moderate AIS scores were predominant (75% mild, 19.5% moderate), further supporting this demographic profile.

### 4.1. AIS and Associated Variables

Three variables evaluated in the current research were found to have significant associations with the AIS. These were the Modified Marshall Score, the education level, and the type of injury.

Building on these findings, the relationship between AIS and the Modified Marshall Score is important. Both scales are commonly used in clinical and research settings for TBI [34,35,36]. 

Furthermore, there was a significant association with the type of injury. Penetrating or crush injuries more often result in more severe trauma and higher AIS scores, a result which is similar to other literature [37]. 

In addition, the level of education was also significantly associated with AIS (*p* = 0.020). Previous research [38,39] shows that individuals with lower education levels may have reduced access to preventive care. They may also be more likely to live or work in environments with a higher risk of injury. 

Finally, no significant associations were found between AIS and other sociodemographic factors. This includes sex (contrary to other studies in the literature that show that female patients usually suffer less severe TBIs that need fewer ICU care days [40,41]), marital status (similar to other research [42]), residence, or employment status (even though there is research that demonstrated that employment status at the time of the injury influences patient outcomes [43]). 

### 4.2. ISS and Associated Variables

Contrary to AIS, ISS was significantly associated with higher number of variables. Specifically, significant differences in ISS were found for variables such as Modified Marshall Score, type of injury, employment status, cause of injury, and place of injury. Some of these findings are expected: for example, more severe CT findings (higher Modified Marshall Scores), penetrating or crush injuries, and road traffic accidents understandably lead to higher ISS scores. This fact was also studied and underlined in previous research papers [44,45].

Building on these findings, the importance of employment status may indicate different exposure to injury risks or differences in physical activity levels across employment groups. In the current sample, there was a high number of retired individuals. Therefore, future studies including an active working population may be needed to fully understand the impact of employment status on the risk of trauma and its correlation with the ISS. Previous research has focused more on the effect of this variable on patient rehabilitation and employment status following the trauma [46,47,48,49]. Similarly, the association between ISS and place of injury also deserves attention. Injuries sustained at home or in the street may reflect different risk environments, potentially influenced by age, comorbidities, or external hazards.

In contrast to the above associations, neither age nor length of hospital stay was significantly correlated with ISS or AIS. Although patients with severe AIS scores had a longer hospital stay (12.9 days), this difference was not statistically significant. Similarly, the weak and non-significant correlations between ISS and both age and length of stay suggest that other factors may play a more dominant role in determining clinical outcomes post-injury. This finding is contrary to other studies [50,51]. 

The only significant difference in ISS observed through Wilcoxon testing was between male and female patients. Males experienced higher severity scores. This aligns with the literature indicating that males are more likely to engage in high-risk behaviors and suffer more severe TBIs [41,52,53]. The absence of a corresponding AIS difference suggests that sex-based disparities may manifest more clearly in overall injury severity than in isolated head injury assessments. ISS includes injuries across all body regions, and women and men might experience different patterns of polytrauma, even when both suffer head injuries. This finding might also suggest that ISS is better suited for mortality prediction or triage, while AIS might be more useful for targeted evaluation of the brain. 

### 4.3. Strengths and Limitations

The current research presents a multi-center analysis using retrospective data from two Eastern European hospitals. This approach enhances the generalizability of the findings and may help policy and healthcare planning in this region. The inclusion of both the AIS and ISS as primary evaluators enables an assessment of TBI severity from both anatomical and systemic perspectives.

Moreover, this study examines a wide range of variables and their impact on TBI severity. These variables include sociodemographic, clinical and injury-related factors. The study highlights both significant and non-significant associations. This contributes to the understanding of factors influencing TBI outcomes and lays the foundation for future research.

The current research also has several limitations. The total sample (*n* = 164) is relatively modest. This is especially true for the severe AIS group (*n* = 9), which may reduce the power to detect differences and limit generalizability across injury severities. Additionally, the data comes from two urban Eastern European hospitals. This may not reflect practices used in rural areas. 

We do not have rater data, so we cannot report inter-rater reliability. This limitation might bias our results. Additionally, we excluded GCS and the Glasgow Outcome Scale (GOS) from the analysis to maintain a larger sample size. However, future research should include them to obtain a more comprehensive picture of the pathology. Although comorbidities were recorded and categorized, their potential impact on the ISS and the AIS was not investigated thoroughly. Even though most patients had none or a maximum of one associated comorbidity, no multivariate analyses were performed to evaluate them as confounding or modifying factors due to the fact that regression-based assumptions were not met. Given the established association between comorbidity burden and TBI outcomes [54,55], this represents a missed opportunity to contextualize the findings better. 

There is also a need for future research regarding functional outcomes and long-term recovery. These aspects were not evaluated in this study. 

## 5. Conclusions

The current study presents correlation and group comparison results between TBI severity, measured by AIS and ISS, and various clinical and sociodemographic factors. While AIS is more important for isolated brain injuries due to the fact that it offers a focused evaluation of injury severity within a specific region, ISS reflects the overall systemic burden. Future studies with longitudinal outcomes are needed to fully understand its complexity. Our findings suggest that these scoring systems reflect different dimensions of trauma and should be seen as complementary rather than interchangeable. These results are especially relevant for Eastern European populations and should guide adaptations in risk stratification, trauma triage, and health policy planning. 

Future studies should integrate detailed comorbidity profiles and long-term outcomes and examine trauma system disparities across Europe.

## Figures and Tables

**Table 1:** Descriptive statistics for 164 TBI patients.

		*n*, %
Sex		
	Female	56 (34.1)
	Male	108 (65.9)
Education		
	High school (9–12 years)	42 (25.6)
	No formal education	4 (2.4)
	Primary school (1–4 years)	6 (3.7)
	Secondary school (5–8 years)	18 (11.0)
	University (13+ years)	69 (42.1)
	Unknown	25 (15.2)
Employment type		
	Employed	36 (22.0)
	Other	3 (1.8)
	Retired	98 (59.8)
	Unemployed	12 (7.3)
	Unknown	15 (9.1)
Marital status		
	Living together	4 (2.4)
	Married	73 (44.5)
	Separated	4 (2.4)
	Single	27 (16.5)
	Unknown	30 (18.3)
	Widowed	26 (15.9)
Residence		
	Rural	43 (26.2)
	Urban	121 (73.8)
Place of injury		
	Home	100 (61.0)
	Other	15 (9.1)
	Sports facility	2 (1.2)
	Street	44 (26.8)
	Work	3 (1.8)
Cause of injury		
	Aggression	5 (3.0)
	Fall	125 (76.2)
	Other	6 (3.7)
	Road traffic accident	28 (17.1)
Type of injury		
	Closed	153 (93.3)
	Crush	7 (4.3)
	Penetrating	4 (2.4)
AIS		
	Mild AIS—2	123 (75.0)
	Moderate AIS—3	32 (19.5)
	Severe AIS—4–5	9 (5.5)
Modified Marshall Score		
	I	12 (7.3)
	II	133 (81.1)
	III	8 (4.9)
	IV	8 (4.9)
	V	3 (1.8)
Comorbidity categories	Zero	35 (21.3)
	One	45 (27.4)
	Two	30 (18.3)
	Three	19 (11.6)
	More than three	32 (19.5)
	Unknown	3 (1.8)

**Table 2:** Descriptives for age and length of stay by AIS category.

AIS Category	Age (Mean, SD)	Length of Stay (Mean, SD)
Mild AIS—2	65.1 (SD: 20)	8.40 (SD: 5.06)
Moderate AIS—3	63.4 (SD: 19.6)	9.59 (SD: 6.56)
Severe AIS—4–5	69 (SD: 16.7)	12.9 (SD: 6.97)

## Data Availability

The data presented in this study are available on request from the corresponding author. The data are not publicly available due to privacy or ethical restrictions.
